# Supplementary figures and images for: Genetic Modulation of Initial Sensitivity to Δ9-Tetrahydrocannabinol (THC) Among the BXD Family of Mice
Source: Front Genet. 2021 Jul 23;12:659012. doi: 10.3389/fgene.2021.659012 (PMC8343140; doi:10.3389/fgene.2021.659012)

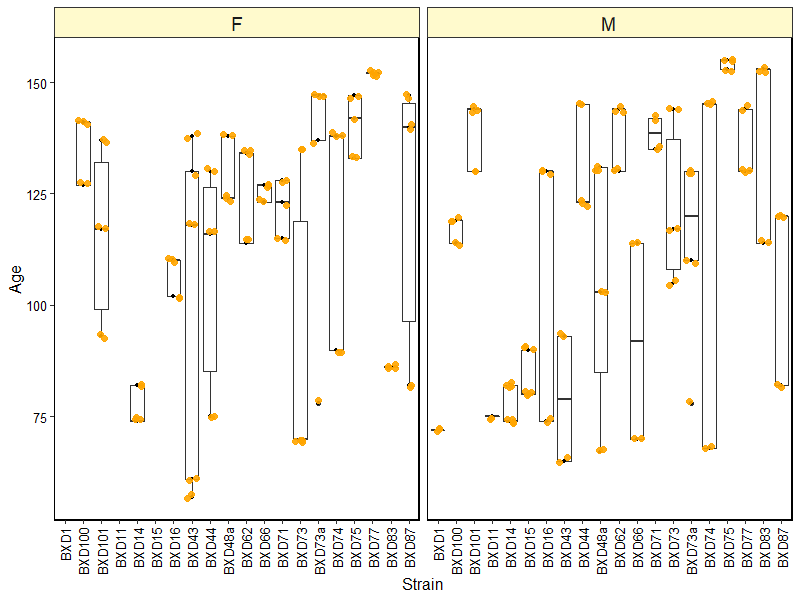

Supplement: Supplementary Figure 1 — Age distribution among BXD subjects. Box plot for each strain and sex is shown. Median indicated by horizontal line. Interquartile range (IQR, 25th to 75th percentiles) represented by box. Whiskers indicate maximum (75th percentile + 1.5 × IQR) and minimum (25th percentile + 1.5 × IQR) range. Black points indicate outliers. Ages of BXD subjects are superimposed on the box plot as orange points. [file Image_1.tiff]

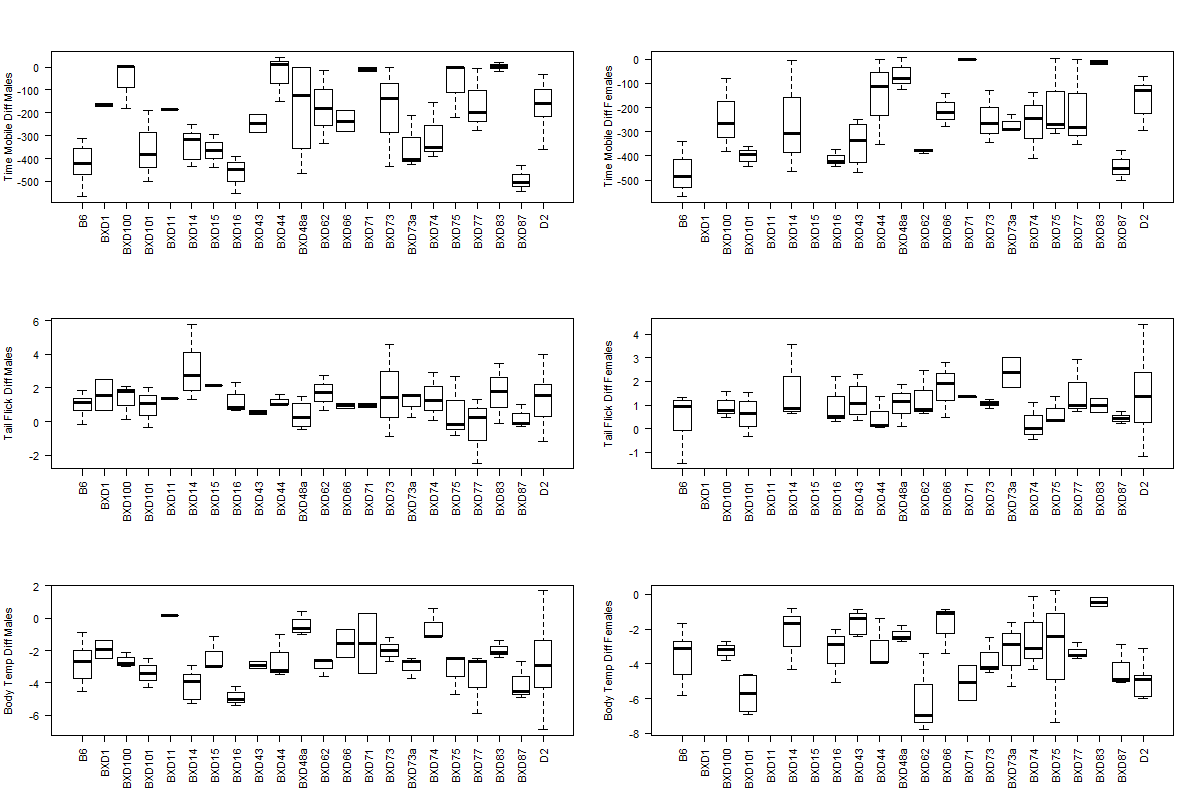

Supplement: Supplementary Figure 2 — Distribution of initial response traits among B6, D2, and their recombinant inbred BXD progeny. Strains are shown on the x-axis and the y-axis shows initial response to THC treatment (difference between baseline day 0 and day 1). Male and female responses for each trait are shown in the left and right columns, respectively. [file Image_2.tiff]

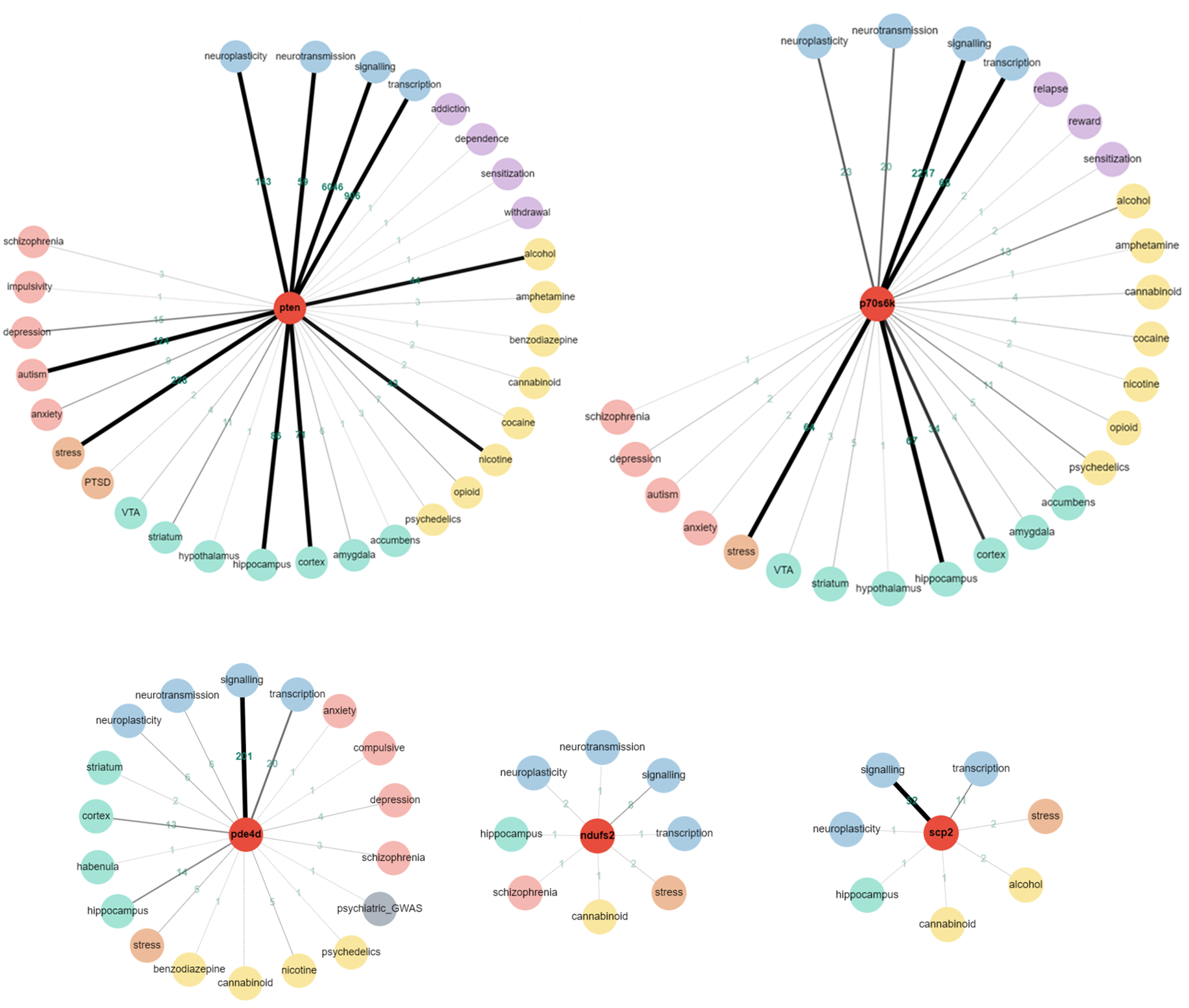

Supplement: Supplementary Figure 3 — Associations between QTGs and signaling, brain region, addiction, and psychiatric disease terms from the GeneCup Resource. The center node (red) represents the QTG. Terms are shown as nodes (colored by group: blue for signaling terms, yellow for drug terms, purple for addiction terms, green for brain region terms, brown for stress terms, and orange for psychiatric terms) radiating around the center node. Edges represent literature associations from PubMed and the edge thickness increases as the number of associations increases. For Pde4d, association to the cannabinoid term is indirect (dashed line) and is linked to PDE4 activity and the edge (de Jong et al., 2015) connected to the psychiatric_GWAS term is for an association (p = 5E-8) between variants in the human gene (rs159497) and forced expiratory volume in 1 s (occupational environmental exposures interaction). [file Image_3.tif]
